# Examining the sustainment of the Adolescent-Community Reinforcement Approach in community addiction treatment settings: protocol for a longitudinal mixed method study

**DOI:** 10.1186/s13012-014-0104-1

**Published:** 2014-08-13

**Authors:** Sarah B Hunter, Lynsay Ayer, Bing Han, Bryan R Garner, Susan H Godley

**Affiliations:** RAND, 1776 Main Street, Santa Monica, 90407-2138 CA USA; RAND, 1200 South Hayes Street, Arlington, 22202 VA USA; Chestnut Health Systems, 448 Wylie Drive, Normal, 61761 IL USA

**Keywords:** Sustainment, Evidence-based treatments, Adolescent substance use treatment, Mixed methods, Longitudinal data analyses

## Abstract

**Background:**

Although evidence-based treatments are considered the gold standard for clinical practice, it is widely recognized that evidence-based treatment implementation in real world practice settings has been limited. To address this gap, the federal government provided three years of funding, training and technical assistance to 84 community-based treatment programs to deliver an evidence-based treatment called the Adolescent-Community Reinforcement Approach (A-CRA). Little is known about whether such efforts lead to long-term A-CRA sustainment after the initial funding ends.

**Methods/Design:**

We will use a longitudinal mixed method data analytic approach to characterize sustainment over time and to examine the factors associated with the extent to which A-CRA is sustained. We will use implementation data collected during the funding period (*e.g*., organizational functioning, staff certification rates and penetration) and supplement it with additional data collected during the proposed project period regarding implementation quality and the hypothesized predictors of sustainment (*i.e*., inner and outer contextual variables) collected over three waves from 2013 to 2015 representing program sustainment up to five years post-initial funding.

**Discussion:**

Gaining a better understanding of the factors that influence the evidence-based treatment sustainment may lead to more effective dissemination strategies and ultimately improve the quality of care being delivered in community-based addiction treatment settings.

## Background

Numerous interventions for adolescent substance use disorders (SUDs) have been developed, tested and supported by empirical evidence, yet of the two million 12- to 17-year-olds in need of SUD treatment, only about 8% actually receive it [1]. Providing high quality care to those youth who access addiction treatment can mitigate the adverse consequences of substance use, including both short and long-term violence, accidents, disease, and criminal behavior [1,2]. Ensuring the provision of quality care also can bolster community confidence in treatment: this, in turn, may lead more families, courts and schools to refer youth to treatment and help ensure that adolescents in need of care receive it.

One strategy policymakers use to ensure high quality treatment is to offer discretionary monies that encourage community-based programs to adopt treatment protocols deemed efficacious in experimental settings (*i.e*., evidence-based treatments or EBTs). For example, government agencies such as the Substance Abuse and Mental Health Services Administration (SAMHSA) have offered discretionary grant funding in order to help facilitate EBT implementation. In one of the largest such efforts to date, the SAMHSA’s Center for Substance Abuse Treatment (CSAT) provided over 80 million dollars to support community-based SUD treatment organizations to implement the Adolescent Community Reinforcement Approach (A-CRA; [3]), an EBT that has yielded positive outcomes in relation to adolescent alcohol use, mental health, and social functioning in three randomized controlled trials (RCTs; [4-6]). These discretionary grants provided on average $300,000 annually for approximately three years to support local implementation and evaluation including a multi-day A-CRA training, technology-assisted performance feedback, and a standardized certification process for both clinicians and supervisors provided by the treatment developers.

While EBT dissemination using intense support from treatment developers has been shown to improve implementation quality by clinicians in private practice [7,8] and those who work in community-based organizations [9,10], little is known about: a) the extent to which such federal grant initiatives lead to sustained EBT implementation and b) the factors that are associated with sustained implementation [11-14]. To understand A-CRA sustainment, that is ‘the continued use of an innovation in practice’ [15], we developed a measure consistent with Fixsen *et al*.’s [11] and Scheirer and Dearing’s [16] definition of implementation quality by assessing both the organizational supports and delivery components of the treatment. Understanding EBT sustainment using both factors has not been well-utilized in previous sustainment research [17]. To date, most reported program sustainment studies have relied on self-reports rather than more objective measures of implementation quality.

There is a lack of empirical evidence on the factors that predict EBT sustainment, and this study is uniquely positioned to study several factors that have been theorized to be related to sustainment. When planning this study in early 2009, we turned to the public health literature on program sustainability to identify factors to examine. We discovered that conceptual frameworks and models of change have been slow to develop, partially due to the diversity in the definition of terms to characterize program sustainment [17-19]. We reviewed literature regarding the seminal work on innovation diffusion in organizations [20] and its application to health service organizations [21], the uptake and implementation of evidence-based practices in medical, mental and public health contexts [11,18,22], organizational change and the adoption of EBTs within SUD treatment programs [23,24], and social or health service program sustainment [25-28]. In reviewing this wide spectrum, we identified four main factors theorized to influence program sustainment: a) the broader community environment, external to the organization implementing the EBT; b) the level of implementation during the funded period; c) factors within the organizational setting, such as leadership support; and d) intervention/innovation characteristics. These factors are consistent with Damschroder and colleagues’ Consolidated Framework for Implementation Research (CFIR), a conceptual approach that is based on a systematic review of the implementation literature published soon after we conceptualized our study [29].

In the CFIR model, the broader community environment is considered the ‘outer context’, that is, those factors outside of the organization under study that influence implementation and sustainment. In this study, we examine policy, regulatory, or fiscal facilitators or barriers, such as whether providers are reimbursed for providing the EBT to their clients [11], community leader involvement [26,30], and the degree of community need for the treatment [31].

The level of implementation refers to how well an EBT was delivered during the funding period. In this study, we assess level of implementation in reference to employing clinical and supervisory staff who have demonstrated competency through the certification processes and an organization having recruited and treated a sufficient number of participants during the initial implementation period, also termed as ‘penetration’ [32,33]. Integral to Rogers’ [20] innovation diffusion theory is that the implementation experience will have a large influence on sustainment. Poor implementation may lead to ‘discontinuance’ (*i.e*., rejection) of an innovation. We have available participant process (*e.g*., treatment initiation, engagement, satisfaction) and outcome data to help explicate implementation during the funding period. Opinions are mixed as to whether these data will help predict level of sustainment [34]. Intuitively, it is sensible to expect that the extent to which clinicians are able to engage participants in an intervention or participants are able to achieve a treatment’s objectives would be related to sustainment. However, such factors as external (*i.e*., community) or internal (*i.e*., organizational) support may trump any efforts to sustain an effective EBT. Therefore, we examine these issues in our study.

Factors within the organizational setting refers to characteristics of the ‘inner context’ [29], such as institutional (*e.g*., leadership support, resources within the organization for EBT delivery), organizational climate [35], and staff attributes (*e.g*., presence of a program champion, motivation and skills to deliver the EBT). Although studies have shown that staff turnover is not predictive of sustainment [17], we believe that with a complex treatment like A-CRA, attrition of trained staff will impede sustainment. Intervention characteristics, such as community-based providers’ perceptions of the treatment, may influence whether it is sustained [20]. It is not known whether program leadership will still be in support of the treatment at the end of the grant period and whether staff will be motivated and trained to deliver it, so we plan to assess these factors in this study.

### Relationships among variables

It is important to acknowledge that the four factors described above do not operate in isolation from one another [22]. Researchers have emphasized the dynamic context of the community in fostering sustainment, whereas both Gruen *et al*.’s [25] and Shortell’s [22] work emphasized the interaction between components (*e.g*., tailoring a treatment to organizations and the community context) and over time (*e.g*., integrating the treatment within existing structures). To better address this point, we will assess these factors through multiple methods across time, including qualitative semi-structured interviews and standardized survey questions that will help us better ascertain the association among these factors. For example, we will ask staff about how the treatment was or was not integrated into their existing organization and whether adaptations were made to the treatment to make it feasible to continue its delivery without the support provided during the funding period.

### Previous studies

It has been historically difficult to study EBT sustainment. After initial funding for EBT implementation ends, resources are seldom available to continue studying implementation [17]. Therefore, most knowledge about sustainment relies on anecdotal evidence, case studies, or highly controlled experiments that have limited external validity [36]. Recently there have been increased attention to assessing program sustainment [16,37,38], but little research has been published in the SUD treatment field. An exception is the work examining pharmacotherapy treatments [39-43]. Behavioral treatments, however, are much more likely to be used in SUD treatment settings [44], and the examination of behavioral treatments warrants different approaches than medication treatments, as the resources needed to implement a medication regime differ from a behavioral treatment. For instance, access to physicians is critical to medication regimes [39], but not to the implementation of behavioral interventions. Furthermore, previous studies have relied on self-report data from program administrators to characterize sustainment. In this study, we plan to determine which factors lead to A-CRA sustainment using more complex implementation quality measures.

In sum, this study will address an important gap in implementation research by examining whether and to what extent an EBT is sustained in usual care practice settings after initial support ends. Additionally, this study will be able to assess factors that predict sustainment because of the implementation and organizational data that have been collected during the funding period and the longitudinal study design that examines program sustainment over a relatively large sample of organizations.

### Study aims and hypotheses

The first study aim is to characterize levels of A-CRA implementation longitudinally for 84 programs that received federal funding to implement A-CRA. In doing so, we will identify which aspects of A-CRA are sustained, innovations in its implementation, and potential facilitators and barriers to implementation. We will also characterize the trajectory of implementation quality among the 84 programs over time. We hypothesize that substantial changes in implementation quality will occur after funding ends. Specifically, we expect to observe an overall decrease in sustainment over time.

The second study aim is to empirically evaluate factors that predict the degree to which programs sustain A-CRA. We hypothesize that the extent to which A-CRA is sustained will be associated with community/outer context (*e.g*., reimbursement for services), organizational/inner context factors (*e.g*., presence of trained supervisors at the end of the funding phase), and intervention-specific characteristics (*e.g*., staff support for A-CRA).

## Methods/Design

### Study context and sample

#### Federally funded A-CRA implementation initiatives

This project examines A-CRA sustainment among SUD treatment programs funded between 2006 and 2010 (see Table 1). During that period, there were four program cohorts funded by the SAMHSA/CSAT called the ‘Assertive Adolescent Family Treatment’ initiative in 2006, 2007, 2009 and 2010 (*e.g*., see http://www.samhsa.gov/Grants/2009/ti_09_002.pdf). For these initiatives, the grantee was required to utilize A-CRA as the treatment regime. In addition, other funding opportunities were offered during this period by the SAMHSA including the ‘Juvenile Drug Court’ and ‘Juvenile Drug Treatment Court’, the ‘Offender Reentry Project’, and the ‘Targeted Capacity Expansion’ initiatives (*e.g*., see http://www.samhsa.gov/grants/2013/ti-13-007.aspx). For these initiatives, the grantee was required to identify an evidence-based treatment, and several of the funded organizations selected A-CRA and therefore were included in our study sample.

**Table 1:** **Number of grantees by the different A-CRA funding mechanisms**

	**No AAFT**	**AAFT1**	**AAFT2**	**AAFT3**	**AAFT4**	**AAFT1&AAFT3**	**AAFT1&AAFT4**	**AAFT2&AAFT3**	**AAFT2&AAFT4**	**AAFT3&AAFT4**	**AAFT1, AAFT3&AAFT4**
JDC	3	0	0	0	0	0	0	0	0	1	0
JTDC	6	1	0	0	0	0	0	0	0	2	0
ORP	6	0	0	0	0	0	0	0	0	0	1
TCE	1	1	0	0	0	0	0	0	0	0	0
None of the above	0	6	15	7	25	3	4	1	1	0	0

*Note*. AAFT: Assertive Adolescent Family Treatment; JDC: Juvenile Drug Court; JDTC: Juvenile Drug Treatment Court; ORP: Offender Re-entry Program; TCE: Targeted Capacity Expansion.

#### Treatment programs

The study sample is composed of nonprofit treatment providers located across the country representing 27 states. Using the most recent data available from the N-SSATs [44], 88% of adolescents receive treatment in outpatient settings, and 66% of that treatment is delivered by nonprofit providers, similar to the proposed study sample. The funder also specified that applicants were required to demonstrate: a) program operation in the same geographical location(s) for at least two years prior to the proposed project period; and b) compliance with local and state/tribal licensing, accreditation and certification requirements. These specifications indicated SAMHSA’s intent to build existing SUD treatment program capacity rather than to support new programs. In some cases, the grantee was a non-SUD treatment provider (*e.g*., school, court, community organization) that partnered with an existing SUD treatment provider in order to deliver the services. Many of the programs (n = 15) received more than one grant during the study period.

#### A-CRA implementation

The A-CRA supervisor and clinician training processes were designed based on research findings of the most effective methods for disseminating EBTs [45]. Initially, all attended a 2.5 day training workshop and then, dependent on their role, were required to demonstrate competency delivering the treatment or providing supervision specific to the model. Clinicians recorded actual therapy sessions at their organizations, while supervisors recorded supervision sessions with their clinicians. Using a rating manual, trained coders provided numeric ratings of the recorded sessions accompanied by written feedback to help improve skills [46-48]. For clinicians, competency was determined based on passing scores on the various A-CRA procedures. Supervisors had to demonstrate specific supervisory skills and knowledge of A-CRA during the recorded supervisory sessions, and also the ability to reliably rate the therapy session recordings when compared to the trained coders. Cross-site coaching calls were provided by model experts bi-monthly. Once supervisors achieved certification, they were able to train and certify clinicians at their local site to facilitate sustainment after the federal funding period ended. Since A-CRA is a menu-based treatment with 19 defined procedures, the certification processes were expected to take from six months to a year.

#### Participant recruitment and eligibility criteria

Although the aims and hypotheses of the current study are focused on treatment organizations, it is necessary to collect information from individuals at the funded programs who would be responsible for implementing the treatment. Given our focus primarily on implementation quality, our target population includes clinical supervisors and clinicians responsible for adolescent treatment. In the case that a site no longer has an adolescent SUD treatment program, we attempt to recruit program directors or other administrative staff with knowledge about the organization’s former adolescent SUD treatment program and about how, why, and to what extent A-CRA was or was not sustained after the federal funding ended.

### Data sources

#### Overview

We will rely on two sources of data: a) secondary data on implementation quality and organizational functioning that were collected during the funding period; and b) primary data collected at three time points during the study to measure the extent of sustainment and a number of hypothesized predictor and control variables.

#### Secondary data

Extensive data was collected during the funding period on implementation quality and organizational functioning. Records of the number of clinicians and supervisors trained and certified at each funded program, and the number of sessions and A-CRA procedures delivered to each client were recorded. Data from clinical staff on organizational functioning was also collected. In addition, the number of adolescents that were treated at each funded program, the number of treatment sessions each client received, and the treatment length of stay was recorded. The client-level outcome dataset that contains baseline, 3-, 6-, and 12-month follow-up data on substance use and functioning is available from clients who receive A-CRA at the funded sites. No individual-level identifiers are included in the secondary dataset. This dataset will be useful to help characterize implementation quality during the funding period and serve as predictor variables to test our hypothesis as to whether programs that were able to successfully implement A-CRA during the funding period are more likely to sustain it than programs that were not successful at implementation during the funding period.

#### Primary data

For this project, we will collect data over three regularly scheduled intervals (approximately every nine months) to assess the level of sustainment (*i.e*., our main dependent measure) and hypothesized predictor and control variables. The first data collection occurred within one year of project funding (*i.e.,* Fall 2013). For the majority of the sites (*i.e*., the AAFT4 2010 cohort, n = 34), the primary data will include data from the last quarter of funding and two times following that period (*i.e*., at time of loss of funding, and approximately one and two years later). For the oldest cohort funded in 2006, the project will collect data approximately 48 months post-funding and up to 72 months post-funding.

### Data collection procedures

#### Primary data collection

Primary data collection will consist of telephone interviews, online surveys, and audiotaped treatment sessions from key staff (*i.e*., clinical supervisors and clinicians). Staff from each of the treatment sites that were responsible for A-CRA delivery (or outpatient adolescent treatment in general, if it is reported that A-CRA is no longer being delivered) are being recruited. During the funding period, most programs had two clinicians trained. We used this estimate to budget for data collection and analyses.

All data collection is voluntary and confidential. Our strategy is to use multiple methods (*i.e*., mail, phone, and email) to introduce and remind the participants about the data collection opportunities, consistent with effective tailored survey methods [49]. Prior to the data collection, we explain the purposes of the research through both an email and by phone and then ask for participation in the study. If we are not able to contact by email or phone, the information and request is sent by mail (using FedEx).

#### Interviews

Semi-structured phone interviews with clinical supervisors and clinicians at the funded programs are being conducted by trained field interviewers. The interviews are arranged individually with respondents and last approximately 30 to 60 minutes depending on respondent role and experience with A-CRA. Following standard semi-structured interview protocols, the interviews use open-ended (*i.e*., ‘grand tour’) questions followed by focused, standard probes, such as verification and compare-and-contrast questions [50]. We provide respondents with compensation for the interview and surveys ($50).

#### Surveys

After the semi-structured telephone interview, we ask respondents to complete an online survey to ascertain characteristics of the facility in which the treatment was delivered that will serve as covariates in our analyses (*i.e*., program-specific factors) and other standardized scale measures of organizational or clinical support (see Table 2 for a list of measures). Our experience with data collection suggests that higher survey response rates will be achieved if it is conducted following the interviews, and this is also consistent with recent data collection approaches in this field [51].

**Table 2:** **Predictors of sustainment, measures, and data collection methods**

***Construct***	***Dimension***	***Measurement source***	***Data collection methods***	***Respondent(s)***
**Community/environment factors**				
External support	▪ Funding for intervention-related services	Knudsen 2009 [53]	Interview & Survey	Supervisor
Community participation	▪ Intervention-relevant referrals	Knudsen 2009 [53]	Survey & Interview	Supervisor
	▪ Dissemination efforts and community involvement	Scheirer *et al*. 2008 [17]		
**Implementation during the funding period-related factors**				
Quantity and quality	▪ # of clients served, # of clinicians certified, # of supervisors certified, average # of sessions provided per participant	Archival data	NA	NA
Client outcomes	▪ 12-month recovery status	Archival data	NA	NA
**Organizational factors**				
Organizational type and capacities	▪ Agency type, services offered, payment sources, age of program, client size and composition	N-SSATS [44]; Knudsen 2009 [53]	Interview & Survey	Supervisor
				Supervisor
	▪ Staff levels & qualifications	Knudsen 2009 [53]	Survey	Supervisor
	▪ Staff turnover (*annual rates, key personnel*)		Survey	
	▪ Sustainability capacity	Wash U, 2012 [55]	Survey	Supervisor
Leadership support	▪ Managerial support for EBT	O’Loughlin *et al*. 1998 [26]; Aarons *et al*., 2013 [54];	Survey & Interview	Clinician
	▪ EBT champion(s)			Clinician
		O’Loughlin *et al*. 1998 [26]		
Organizational Readiness to Change	▪ Motivation to change, resources, staff attributes, organizational climate	Lehman *et al*. 2002 [35]	Survey	Clinician
**Intervention characteristics**				
EBT perceptions	▪ Perceptions of the EBT intervention (a)	(a) Steckler *et al*. 1992 [56]; O’Loughlin *et al*. 1998 [26]	(a) Interview & Survey	Clinician (a)
	▪ Attitudes toward the EBT intervention (b)			Supervisor & Clinician (b, c, d)
	▪ Plans for EBT sustainment or spread (c)	(b) Lin *et al*. 2005 [57]; O’Loughlin *et al*. 1998 [26]	(b) Interview & Survey	
	▪ Perceived implementation facilitators and challenges (d)	(c & d) Scheirer *et al*. 2008 [17]	(c & d) Interview	

#### Treatment sessions

Following the interview, participants that report providing direct services to youth are asked to submit recorded therapy sessions so that treatment fidelity can be assessed. A technology-assisted system similar to one used during the funding period is being used. This system allows clinicians to upload digitally recorded audiotaped clinical therapy sessions, and its secure role-based system limits access to only one’s own recordings. Clinicians are provided with instructions to obtain client consent to record the treatment sessions. Respondents who are asked to provide recorded therapy sessions receive an additional $50 incentive for this data collection component.

### Study measures

#### Sustainment

We are studying the extent to which A-CRA is sustained by assessing implementation quality. Consistent with Fixsen *et al*.’s [11] and Scheirer and Dearing’s [16] definition of implementation, we operationalized implementation quality by evaluating the functional components of the treatment, including the organizational supports of training and monitoring (*i.e*., supervision), along with treatment quality (treatment fidelity).

The organizational supports for A-CRA delivery includes eight elements: a) clinical knowledge in A-CRA, b) execution of a planned number of treatment sessions at or above the minimum required by developer, c) presence of a certified clinician(s) delivering A-CRA, d) presence of a certified A-CRA supervisor, e) bi-weekly supervision that is aligned with the developer manual, f) supervision that includes review of recorded sessions, g) a training process that meets quality requirements required during implementation, and h) a clinical certification process that mirrors one used during implementation. These elements are based on those that were used during the federal funding period to monitor implementation. Treatment fidelity is assessed via recorded therapy sessions. Clinicians that are currently delivering adolescent SUD treatment services will be identified and asked to upload a sample for assessment. Fidelity ratings will be completed by an expert rater under the supervision of the treatment developer from Chestnut Health Systems, using the A-CRA rating manual [48]. The manual provides operational definitions of each rating option (on a 5-point scale) for each A-CRA procedure. We will compute an average treatment fidelity rating for each clinician based on the ratings across the submitted session. We will then average the fidelity ratings across clinicians at an organization to obtain a program-level treatment fidelity rating. If we learn that one clinician at a site delivers treatment to substantially more clients than another clinician, then we will consider weighting the fidelity data to adequately represent this difference in treatment delivery among clinicians at a program. For this measure, we will create an additive measure by summing up scores on each of the implementation quality components to derive a site-level sustainment score.

#### Predictor measures

We selected measures that have demonstrated good reliability and validity in previous studies. However, given that this area of study is relatively new, we also had to adapt existing measures for the purposes of the current study, and develop measures when necessary. The psychometric properties of all measures, including new or adapted ones, will be examined within our sample using exploratory and confirmatory factor analysis. Our predictors of sustainment measures are shown in Table 2.

#### Community environment factors

Supervisors are asked about local, state and federal support (external support) since the end of federal funding. In these contexts, community participation typically encompasses the provision of referrals to receive care. We ask supervisors about the main sources of A-CRA referral and of their treatment program overall. We ask this in both the interview and survey to capture: a) descriptive information about the reasons for the different referral sources and document any changes over time to help address community need, and b) systematically collect comparable information across the programs on their main sources of referral. We also ask questions related to dissemination efforts and community involvement in the treatment to capture efforts made by the organization to build community support for their program [17]. Supervisors will be asked about the introduction and continuation of other adolescent SUD treatment providers in their area to assess community need changes.

#### Implementation-related factors

A number of variables will be used to characterize implementation during the federal funding period. These include: the number of adolescents treated, the average number of treatment sessions each client received, the average treatment length of stay, and the number of supervisors and clinicians achieving supervisor and clinician certification. We will also use client outcome data collected during the period. Specifically, data were collected using the Global Appraisal of Individual Needs (GAIN; [52]). These data will be used to examine the proportion of clients in recovery 12 months after treatment intake. Data from over 6,000 adolescents receiving A-CRA is available. We anticipate that clients will have the following characteristics based on the current data available: 72.8% male, 31.7% Caucasian, 30.0% Hispanic, 17.5% African American, 20.8% Other. Average age is 16.2 years, 84.4% are attending school, and 51.0% are from a single parent household. Clinically, 90.3% have used alcohol in their lifetime (76.4% to intoxication) with a median age of first use of 13.0 and a median of 3.0 years of use. During the 90 days prior to intake, 57.8% had consumed alcohol (40.7% to intoxication). The latter includes 76.4% consuming a peak of more than five standard drinks. Over 86% of alcohol use was in combination with marijuana or another drug.

#### Organizational setting factors

To assess staff levels and qualifications, we ask supervisors about workforce characteristics (*e.g*., the number of licensed and certified staff, and staff to client ratio) using questions from a survey of adolescent treatment providers [53]. This information will help us determine whether program composition affects A-CRA sustainment. Supervisors are asked about the annual turnover rates for administrative and clinical staff. We also will use turnover information from the implementation period from archival sources. We ask clinical staff about their perceptions of leadership support in the interview. In addition, we included a recently published scale in the online survey designed to assess leadership support of EBT implementation [54].

#### Organizational climate

We use items from the Organizational Readiness for Change (ORC; [35]), which asks about motivation for change, resources, staff attributes, and organizational climate. ORC data was also collected from staff at each of the CSAT-funded organizations during the funding period.

#### Organizational type and capacities

As part of the survey, supervisors are asked questions about the composition of the organization (*e.g*., type of agency [*i.e*., substance abuse treatment services, mental health services, general health care, other], types of payment accepted [*e.g*., Medicaid, SCHIP, private health insurance], other funding sources [federal, state, local government and/or foundations], and clients [age ranges]). These organizational questions are similar to those asked either by National Survey of Substance Abuse Treatment Services [44] and Knudsen [53], a survey specifically designed for adolescent treatment providers. Also, a recent measure developed to assess program sustainability capacity (Washington University, 2012 [55]) is included.

#### Intervention characteristics

##### Perceptions of the A-CRA intervention

We assess A-CRA perceptions in a number of ways. In the interviews, we ask an open-ended question about what respondents thought about A-CRA, which will then be coded for analyses. As part of the survey*,* modified items from O’Loughlin *et al*. [26] are used to assess perceived compatibility, difficulty and effectiveness. Following Rogers’ theory of innovation diffusion, we also employ Steckler *et al*.’s [56] relative advantage and complexity scales, shown to have high reliability (alphas = 0.88 and 0.83, respectively).

##### Attitudes toward A-CRA intervention

We modified survey items developed by Lin *et al*. [57] that assess expectancies, instrumentality and valence associated with implementing a new initiative. These scales have been shown to be related to organizational commitment to implementing new healthcare initiatives.

##### Plans for sustainment and spread

Feinberg *et al*. [58] found that planning for sustainment was predictive of coalition sustainment. We ask supervisors about their organization’s plans to sustain A-CRA.

##### A-CRA facilitators and barriers

We use items developed by Scheirer *et al*. [17] to ask all supervisors and clinicians about the factors that facilitated and impeded A-CRA delivery regardless of whether they report sustainment or not. If staff report that they are no longer delivering A-CRA, we ask about the reasons for stopping it (*e.g*., ‘When did you stop delivering A-CRA?’, ‘What were the main reason(s) that you stopped delivering it?’) and ask those that report sustainment the reasons that helped them maintain it (*e.g*., ‘What has helped you maintain A-CRA since the loss of CSAT funding?’). We use these data to meet the first study aim.

### Analytic plan

This study involves the use of both qualitative and quantitative analytic methods. The first aim of describing sustainment and the associated innovations, facilitators and barriers will be addressed primarily with qualitative methods. We employ multiple strategies to avoid bias in the analysis and interpretation of the qualitative data by including the use of a multidisciplinary team (rather than a single observer) to collect and analyze the data. In addition, standardized instruments along with semi-structured interview protocols are used to decrease the possibility of bias.

Raw data will be cleaned and descriptive statistics will be conducted at first to check the data quality and integrity for every variable in the raw data. In case of missing information on the quantitative measures, multiple imputation and nonresponse weights will be implemented [59]. All data will be aggregated to the program by time level for subsequent analyses. We recognize that our study design includes data from different time points based on when the organization lost or will lose funding and our proposed data collection period (2013 to 2015). The mixed model can handle these unsynchronized longitudinal measurements so that we can consistently estimate the association between the predictors and the proposed outcomes.

Next, preliminary exploratory analyses will estimate the unadjusted mean trajectories of sustainment outcomes over time among all sites as well as for each site by growth curve models [60] to address the goals of the first study aim. A cluster analysis will [61] identify potential groups of sites where sites in a group will have relatively similar trajectories. If such groups exist, descriptive statistics will be conducted to summarize and compare the mean characteristics of all groups.

In order to address the second study aim, hierarchical mixed-effect models will be applied to estimate the effects of predictors on the sustainment outcomes. These models will include the fixed effects for all study factors and covariates as well as the random effects for site-level temporal trends. Cohorts of sites will also be controlled to account for differences among funding waves. Fixed effects consist of both static factors, *e.g*., implementation quality during funding period, and time varying factors, *e.g*., external funding support. Correlation analyses among predictors and the variance inflation factors will be used to evaluate the potential multicollinearity in fixed effects. If necessary, a model selection procedure such as backward-elimination regression will be applied to drop some correlated predictors. Statistical significance of a predictor will be tested by Wald’s test (z-test) or the likelihood ratio test (chi-squared test) after fitting the final mixed-effect models. F-tests will also be conducted to test multiple factors simultaneously, *e.g*., external supports received during the implementation period (static) and after the implementation period (time-varying).

### Estimated sample size and statistical power

We estimate the study will have 64 programs for 3 time points, which means a response rate of roughly 80% at the site level. Assuming this sample size and an intra-class correlation within each site of 0.1, we estimate that the proposed sample size is sufficient to detect an MDE of approximately 0.45 standard deviations between two levels of a dichotomous predictor, *e.g*., the program type. For a continuous predictor, this sample size is sufficient to detect a correlation coefficient of approximately 0.34. These effect sizes are considered small to medium [62]. We expect better power in our analysis, since the mixed-effect models are more powerful than bivariate analysis in the simplified power calculation.

### Trial status

Study protocols have been approved by the institutional Internal Review Board (IRB) RAND Human Subjects Protection Committee (HSPC). At the time of submission of this manuscript (July 2014), the first wave of data collection has been completed.

## Discussion

### Contributions to science and practice

The current study will substantially add to the implementation science field as well as offer policy and clinical practice recommendations to guide future efforts to diffuse EBTs to address behavioral health concerns. While efficacious interventions are critical for improving treatment quality, they hold little value without attention to their implementation and sustainment [63]. Little is known about the long-term effectiveness of the large dissemination efforts currently underway to improve adolescent SUD treatment quality. There is already a sizeable amount of infrastructure and data in place to help inform whether A-CRA can be sustained in community practice settings after the initial support for delivery ends. This study will build upon these efforts to improve understanding of EBT sustainment and the factors that influence it. This study represents an important step in increasing treatment quality to address adolescent substance use as well as informing the emerging implementation science field.

### Limitations

Although we have information about client outcomes from the implementation period, we do not have the resources to monitor client outcomes during the sustainment period. Rather, we rely on assessments of treatment penetration and treatment fidelity that theoretically should be related to client outcomes and are important implementation outcomes [32]. A second potential limitation is the representativeness of the study sample and therefore the external validity of the study. In response to this concern, it is important to recognize that the funded programs reflect many of the same characteristics of the most common form of treatment offered in the U.S. (*i.e*., nonprofit, licensed, outpatient services) and range in size and organization type [64]. The federal government has offered discretionary grant funds to support EBT delivery for more than a decade and continues to use this mechanism. Moreover, many foundations and other grant-making organizations offer ‘seed money’ or initial funding to support EBT implementation and expect communities to find other sources of support to continue service delivery. Therefore, we think this ‘natural experiment’ is worthy of study as it will offer many lessons learned for both the implementation research field as well as clinical practice and policy.

## Abbreviations

AAFT: Assertive Adolescent Family Treatment
A-CRA: Adolescent Community Reinforcement Approach
CSAT: Center for Substance Abuse Treatment
EBT: Evidence-based treatment
JDC: Juvenile Drug Court
JDTC: Juvenile Drug Treatment Court
ORP: Offender Re-entry Program
RCT: Randomized controlled trial
SAMHSA: Substance Abuse and Mental Health Services Administration
SUD: Substance use disorder
TCE: Targeted Capacity Expansion
